# Systolic Blood Pressure and Outcomes in Stable Outpatients with Recent Symptomatic Artery Disease: A Population-Based Longitudinal Study

**DOI:** 10.3390/ijerph18179348

**Published:** 2021-09-04

**Authors:** Juan F. Sánchez Muñoz-Torrero, Guillermo Escudero-Sánchez, Julián F. Calderón-García, Sergio Rico-Martín, Nicolás Roberto Robles, M. Asunción Bacaicoa, José N. Alcalá-Pedrajas, Guadalupe Gil-Fernández, Manuel Monreal, on behalf of the FRENA Investigators

**Affiliations:** 1 Department of Internal Medicine, Hospital San Pedro Alcántara, 10003 Cáceres, Spain; juanf.sanchezm@gmail.com; 2 Department of Internal Medicine, Hospital Virgen Del Puerto, Plasencia, 10003 Cáceres, Spain; guille_sanchez0912@hotmail.com; 3 Department of Nursing, Nursing and Occupational Therapy College, University of Extremadura, 10003 Cáceres, Spain; jfcalgar@unex.es; 4 Department of Nephrology, Hospital Infanta Cristina, 06080 Badajoz, Spain; nrrobles@unex.es; 5 Centro de Salud Manuel Encinas, 10001 Cáceres, Spain; masuncionb8@gmail.com; 6 Department of Internal Medicine, Hospital Comarcal Valle de los Pedroches, 14400 Pozoblanco, Spain; jnalcala58@hotmail.com; 7 Department of Nursing, Faculty of Medicine, University of Extremadura, 06080 Badajoz, Spain; ggilfer@unex.es; 8 Department of Internal Medicine, Hospital Germans Trias i Pujol, Badalona, 08916 Barcelona, Spain; mmonreal.germanstrias@gencat.cat

**Keywords:** systolic blood pressure, outcomes, symptomatic artery disease, ischemic event, death

## Abstract

Objectives: The most appropriate targets for systolic blood pressure (SBP) levels to reduce cardiovascular morbidity and mortality in patients with symptomatic artery disease remain controversial. We compared the rate of subsequent ischemic events or death according to mean SBP levels during follow-up. Design: Prospective cohort study. FRENA is an ongoing registry of stable outpatients with symptomatic coronary (CAD), cerebrovascular (CVD) or peripheral artery disease (PAD). Setting: 24 Spanish hospitals. Participants: 4789 stable outpatients with vascular disease. Results: As of June 2017, 4789 patients had been enrolled in different Spanish centres. Of these, 1722 (36%) had CAD, 1383 (29%) CVD and 1684 (35%) PAD. Over a mean follow-up of 18 months, 136 patients suffered subsequent myocardial infarction, 125 had ischemic stroke, 74 underwent limb amputation, and 260 died. On multivariable analysis, CVD patients with mean SBP levels 130–140 mm Hg had a lower risk of mortality than those with levels <130 mm Hg (hazard ratio (HR): 0.39; 95% CI: 0.20–0.77), as did those with levels >140 mm Hg (HR: 0.46; 95% CI: 0.26–0.84). PAD patients with mean SBP levels >140 mm Hg had a lower risk for subsequent ischemic events (HR: 0.57; 95% CI: 0.39–0.83) and those with levels 130–140 mm Hg (HR: 0.47; 95% CI: 0.29–0.78) or >140 mm Hg (HR: 0.32; 95% CI: 0.21–0.50) had a lower risk of mortality. We found no differences in patients with CAD. Conclusions: In this real-world cohort of symptomatic arterial disease patients, most of whom are not eligible for clinical trials, the risk of subsequent events and death varies according to the levels of SBP and the location of previous events. Especially among patients with large artery atherosclerosis, PAD or CVD, SBP <130 mm Hg may result in increased mortality. Due to potential factors in this issue, Prospective, well designed studies are warranted to confirm these observational data.

## 1. Introduction

Patients with symptomatic artery disease are at increased risk for subsequent ischemic events, and could likely benefit from adequate control of risk factors for atherosclerosis, including hypertension. However, the most appropriate targets for systolic blood pressure (SBP) levels to reduce cardiovascular morbidity and mortality remain controversial [1,2]. Only few studies have been specifically designed to identify the optimal SBP levels after an arterial ischemic event, and most were performed in patients with either cerebrovascular (CVD) or coronary disease (CAD) [3,4,5,6,7,8,9,10,11]. There are scarce data from patients with peripheral artery disease (PAD) [12,13].

The FRENA (Factores de Riesgo y Enfermedad Arterial) registry is a longitudinal, observational and multicenter registry to collect and analyse clinical data, treatment patterns and outcomes in stable outpatients with PAD, CVD or CAD from Spanish hospitals. [14,15,16,17,18,19]. The objective of our study was to evaluate the association of recurrence of subsequent ischemic events (limb amputation, ischemic stroke or myocardial infarction) or death in patients included in the FRENA Registry according to SBP levels during follow-up.

## 2. Methods

The FRENA registry is a real-world cohort of symptomatic arterial disease patients. This is a observational, longitudinal and multicenter study to record clinical, treatment and outcome data of a stable outpatients cohort with CVD, CAD and PAD. The follow-up of the FRENA registry analyzes the possible risk factors of recurrence of subsequent ischemic events or death of the included subjects.

### 2.1. Inclusion Criteria

**Figure 1:**
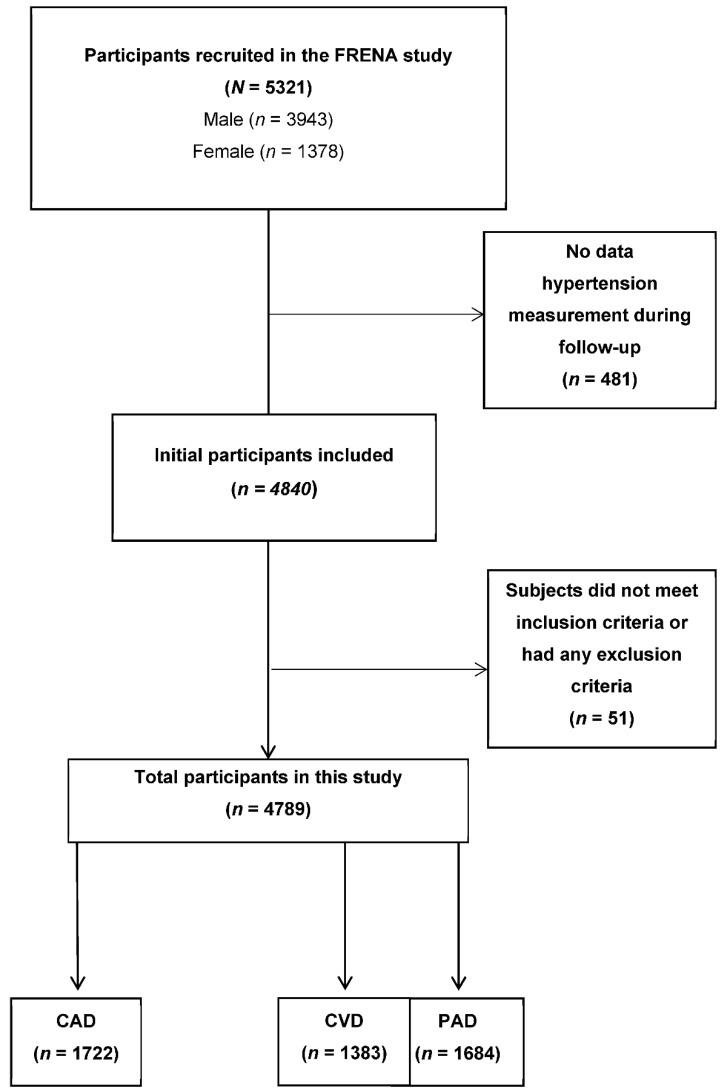
Participant selection process.

Hospitals participating in the FRENA registry prospectively recruited consecutive outpatients with medical history-based symptomatic artery disease with at minimum one recent (less than three months prior to recruitment) episode of PAD (either intermittent claudication with an ankle-brachial index (ABI) < 0.9, previous vascular intervention or limb amputation for PAD); CVD (manifesting as transient ischemic attack or ischemic stroke); or CAD (manifesting as angina or acute coronary syndrome). Subjects with intermittent claudication (ABI ≥ 1.4) were excluded. Exclusion criteria were, if they would not be available for follow-up or if they were currently participating in a clinical trial with blinded therapy. All patients gave written consent to participate in the registry, in accordance with the requirements of each hospital’s ethics committee (Hospital San Pedro de Alcántara (Ref. 14/0189), Hospital Comarcal Valle de los Pedroches (Ref. 14/0032) and Hospital Germans Trias i Pujol (Ref. 14/0524)). Figure 1 shows the participant selection process.

### 2.2. Study Design

BP measurements were conducted with subject seated early in the morning. It involved three measurements of SBP and DBP, and its value was calculated as the mean values of the last two performed using a validated oscillometric device according to the recommendations of the European Society of Hypertension published in 2013 [20]. The major outcomes were the incidence of subsequent ischemic events (either amputation ischemic stroke or myocardial infarction (MI)) and mortality. Outcomes were analyzed into three subgroups according to mean SBP levels during follow-up (<130, 130–140 and >140 mm Hg). Ischemic stroke was considered if the patient had a clinical event, and had a brain MRI or CT that indicated a compatible low-density lesion. Myocardial infarction was identified as the presence of ischemic symptoms in combination with a transient increase of troponin or CK-MB, and/or charasteristic electrocardiogram signs (ST-segment elevation or depression or development of pathologic Q-waves). All outcome data were confirmed by local researchers in the clinical history and were double-checked with the steering committee if it was needed. The Cockcroft and Gault formula was used to calculate baseline creatinine clearance levels.

### 2.3. Follow-up

A clinical history was performed on all patients included in the study. Co-morbid conditions such as history of PAD, CVD, CAD, cancer, diabetes, chronic lung disease, chronic heart failure and smoking status were considered. Then, physical examination was performed including body height and weight, heart rate, electrocardiogram and blood pressure levels on standard conditions, after 5 min of rest. After the baseline visit, subjects were followed-up at 4-month intervals. During these visits, data from physical examination and medical history were registered, including laboratory tests, lifestyle habits, the type, dose, and duration of treatment received, risk factors and clinical outcome. Physicians could use all appropriate medicines, according to their usual clinical practice pattern.

### 2.4. Data Collection

All eligible patients were consecutively registered. Patient identities remained confidential because they were identified by a unique number assigned by the study coordinating center, which was responsible for all data management. Data were recorded on to a computer-based case report form at each participating hospital and submitted to a centralized coordinating center through a secure website. Data quality was routinely checked and electronically reported to identify errors or inconsistencies, which were solved by the local coordinators. Data quality was also controlled through regular visits to participating hospitals by contracted research organizations that verified the medical registers with the data in the website. A full data audit was carried out at regular intervals.

### 2.5. Statistical Analysis

Patients were categorised into three groups according to SBP levels. The SBP < 130 mm Hg group was compared with the SBP groups 130–140 and >140 mm Hg. For this purpose, we used chi-square test (two-side) or Fisher’s Exact Test (two-side) to compare categorical variables and Student’s *t*-test or U-Mann Whitney test for continuous variables. Incidence rates were calculated as cumulative incidence (events/100 patient-years) and compared using the rate ratio (RR) and 95% CI. Associations between the three subgroups of patients and risk of subsequent ischemic events or death were assessed using Cox proportional hazards survivorship model. Hazard ratios (HR) and corresponding 95% confidence intervals (CI) were calculated, and a *p* value < 0.05 was considered to be statistically significant. The variables included as predictors/confounders were: the patient’s age, gender and body mass index, account of chronic heart disease, chronic lung disease, cancer, hypertension, diabetes or dyslipidemia, eGFR *t* presentation, family history of premature coronary artery disease, current smoking habit during follow-up, ABI, mean systolic blood pressure levels and heart rate during follow-up, and the use of beta-blockers, diuretics, calcium antagonists, ACE-inhibitors, angiotensins-II antagonists, oral antidiabetics, insulin therapy, statins anticoagulants or antiplatelets during follow-up. All variables achieving a significance level of *p* < 0.1 in univariate analysis were considered for inclusion in the construction of the Cox model. The data were analyzed using the IBM^®^ statistical program SPSS ^®^ Statistics V.24 (IBM Corporation, Armonk, NY, USA).

## 3. Results

As of June 2015, 4789 patients had been enrolled in various participating Spanish centers. Of these, 1684 (35%) had PAD, 1722 (36%) had CAD and 1383 (29%) CVD. The study included 23,508 measurements of SBP levels during the follow-up period (median, 5 measurements per patient; range, 2–12), with one measurement every 4.80 patient-months on average. Overall, 1664 patients (35%) had mean SBP levels <130 mm Hg, 1302 (27%) 130–140 mm Hg and 1823 (38%) >140 mm Hg.

Patients with mean SBP levels 130–140 mm Hg were slightly older and more likely to be currently smoking, or to have diabetes, CVD or PAD than those with levels <130 mm Hg, but less likely to have CAD or chronic heart disease (Table 1). At baseline, patients with mean SBP levels between 130–140 mm Hg had higher levels of creatinine clearance (CrCl) or cholesterol. They also were more likely to receive diuretics, angiotensin-II antagonists, calcium antagonists, insulin or oral antidiabetics, and less likely to receive beta-blockers than those with mean SBP levels <130 mm Hg. Patients with mean SBP levels >140 mm Hg were also older and more likely to have cancer, diabetes, CVD or PAD than those with levels <130 mm Hg, but less likely to have CAD or chronic heart disease.

Over a mean follow-up of 18 months, 136 patients suffered subsequent MI, 125 ischemic stroke, 74 underwent limb amputation and 260 died (Table 2). Among patients with CAD, those with mean SBP levels >140 mm Hg had a higher rate of subsequent MI (rate ratio (RR): 2.44; 95% CI: 1.45–4.08), ischemic stroke (RR: 13.0 95% CI: 3.10–88.4) or death (RR: 2.99; 95% CI: 1.77–5.06) than those with mean levels <130 mm Hg (Table 2). CAD patients with SBP levels of 130–140 mm Hg also had a higher rate of ischemic stroke (RR: 7.34; 95% CI: 1.55–52.9) or death (RR: 2.99; 95% CI: 1.77–5.06) than those with levels <130 mm Hg. On multivariable analysis however, patients with mean SBP levels 130–140 mm Hg or those with levels >140 mm Hg did not have a significantly higher risk for subsequent ischemic events (Table 3) or death (Table 4).

In patients with CVD, there were no differences in the rates of subsequent events according to mean SBP levels, with the only exception of patients with levels 130–140 mm Hg, who had a lower mortality rate than those with levels <130 mm Hg (RR: 0.47; 95% CI: 0.23–0.91). Multivariable analysis confirmed that CVD patients with mean SBP levels 130–140 mm Hg had a lower risk of mortality than those with levels <130 mm Hg (hazard ratio (HR): 0.39; 95% CI: 0.20–0.77), as did those with levels >140 mm Hg (HR: 0.46; 95% CI: 0.26–0.84).

As for patients with PAD, those with mean levels >140 mm Hg had half the rate of limb amputations (RR: 0.48; 95% CI: 0.27–0.85) and a 3-fold lower mortality rate than those with levels <130 mm Hg (RR: 0.36; 95% CI: 0.23–0.55). Moreover, patients with mean levels 130–140 mm Hg also had a lower mortality rate (RR: 0.48; 95% CI: 0.29–0.78) than those with levels <130 mm Hg. On multivariable analysis, patients with mean SBP levels >140 mm Hg had half the risk for subsequent ischemic events (HR: 0.57; 95% CI: 0.39–0.83) and those with levels 130–140 mm Hg (HR: 0.47; 95% CI: 0.29–0.78) or >140 mm Hg (HR: 0.32; 95% CI: 0.21–0.50) had a lower risk of mortality (Table 4).

## 4. Discussion

In historical cohorts we found an increased rate of subsequent ischemic events and a higher mortality rate in patients with symptomatic artery disease and raised SBP levels [1]. There is extensive evidence on the health benefits of lowering SBP levels in hypertensive patients with established cardiovascular disease [3,4,5,6,7,8,9,10,11]. However, in our cohort of stable outpatients with symptomatic artery disease we failed to confirm any benefit in terms of survival nor in the risk for subsequent ischemic events in patients with SBP levels <130 mm Hg compared to those with higher levels. Unexpectedly, CVD and PAD patients with SBP levels >130 mm Hg during follow-up had less than half the risk of mortality during follow-up. Interestingly, PAD patients with raised SBP levels also had a lower risk for subsequent ischemic events (particularly, limb amputation).

Patients with CAD and raised levels of SBP had a significantly higher non-adjust rate of subsequent ischemic events (subsequent MI, stroke or limb amputation) and a 3-fold higher mortality rate than those with mean levels <130 mm Hg. However, the influence of mean SBP levels on outcome disappeared after adjusting for potentially confounding variables. Our findings are in disagreement with those reported in two meta-analyses [1,21], that suggest some benefits from SBP intensive treatment. However, a 2018 Cochrane systematic review of randomized trials of patients with cardiovascular disease [22] found no changes in total cardiovascular events or mortality for a SBP target less than 135 mmHg. Thus, our experience agrees with the belief that, in hypertensive patients with cardiovascular disease, no net health benefit is derived from reaching SBP levels lower than standard blood pressure target [23,24]. A special issue is the very limited evidence-based data to guide hypertension management in PAD patients. In the Cochrane review [22], only 46 (0.48%) patients with PAD were recruited from 9484 patients with arterial disease. Consequently, there is no evidence to reach conclusions for PAD patients.

**Figure 2:**
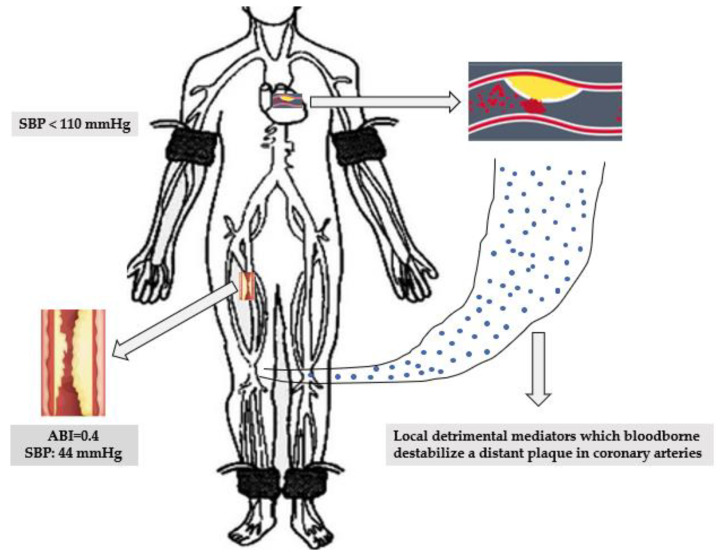
Proposed mechanism of destabilization of a coronary plaque by distal ischemia. A patient with PAD and ABI of 0.4 may have SBP leg lower of 44 mm Hg to a brachial SBP of 110 mm Hg. Local detrimental mediators, free oxygen radicals or inflammatory mediators bloodborne can produce a myocardial event by destabilize a coronary plaque. Abbreviations: ABI, ankle brachial index; SBP, systolic blood pressure.

The optimal SBP levels in PAD patients are controversial, and there are no data for mortality. PAD patients included in the ACCORD [25] and SPRINT [26] trials had contradictory results. However a sub-study of INVEST^26^ and a new recent study [13] found that these patients displayed a J-shape relationship, and patients with levels ≤120 or >140 mm Hg were at increased risk for subsequent events or cardiovascular death, but no all-cause mortality. Our findings show that death was more likely if SBP < 130 mm Hg in patients with PAD or CVD, and these findings disagree with those in SPRINT [26]. However, most patients in this trial had CAD, and the methods of unattended office SBP measurement had generated controversy [27]. Thus, to our knowledge, the decrease in the risk of mortality for patients with CVD or PAD with raising SBP levels during follow-up has not been reported earlier. Potential mechanisms for the inverse correlation between SBP levels and mortality may be attributed to the fact that in patients with high atherosclerotic burden, low SBP levels might decrease perfusion and induce local detrimental mediators which produce harm to distant systemic organs (Figure 2) [28].

Theoretically, similar benefits of reducing SBP could have been expected both in patients with CAD and CVD or PAD. However, this study show that arterial risk factors may have different effect on specific arterial beds. Cholesterol is particularly important in CAD [29], hypertension in CVD [30], and hypertension, smoking and diabetes in PAD [31]. Thus a reduction of SBP could have a different impact on prognosis of subjects with previous vascular event depending on their location.

This longitudinal and observational study has several limitations. Firstly, the subjects included in the FRENA registry were treated according to standard clinical practice, so it was not designed to assess the efficacy and safety of different treatments. The FRENA registry collects clinical data from the natural history of patients who have previously experienced a cardiovascular event, so the design of this study was observational and, therefore, the results indicate association and not causality. However, our data report from real-world clinical situations, generating new hypotheses to help design clinical trials that may explain the causes/effect of strict control of the BPS in these patients. Finally, the results are of interest in that they confirm some other studies that show too low a BP on treatment may be harmful. On the other hand, this challenges other studies and philosophies suggesting “the lower the better”. It is however, difficult to exclude the possibility that a significant number of other harmful conditions contributed to the lower BP.

## 5. Conclusions

In conclusion, this cohort of real-world patients with clinically evident atherosclerotic vascular events suggested that the risk of death varied according to SBP and the location of the previous arterial event. Especially among patients with large-artery atherosclerosis, PAD or CVD, SBP < 130 mm Hg may result in increased mortality. If confirmed by properly designed studies, our findings can have a strong impact on clinical practice.

## Figures and Tables

**Table 1:** Clinical characteristics of the patients according to their mean SBP levels during follow up.

Clinical Characteristics	<130 mm Hg	130–140 mm Hg	>140 mm Hg
*Patients, n*	1664	1302	1823
*Clinical characteristics*			
Mean age (years)	63 ± 13	67 ± 11 ^‡^	69 ± 10 ^‡^
Gender (male)	1306 (78%)	965 (74%) ^†^	1291 (71%) ^‡^
Body mass index (Kg/m^2^)	27 ± 4.4	28 ± 4.2 ^‡^	29 ± 4.5 ^‡^
*Underlying diseases*			
Cancer	79 (4.7%)	71 (5.5%)	122 (6.7%) *
Diabetes	552 (33%)	523 (40%) ^‡^	799 (44%) ^‡^
Current smokers	318 (19%)	326 (25%) ^‡^	394 (22%)
Chronic lung disease	254 (15%)	181 (14%)	251 (14%)
Chronic heart disease	164 (9.9%)	88 (6.8%) ^†^	134 (7.4%) ^†^
*Clinical presentation*			
Coronary artery disease	900 (54%)	442 (34%) ^‡^	380 (21%) ^‡^
Cerebrovascular disease	364 (22%)	439 (34%) ^‡^	580 (32%) ^‡^
Peripheral artery disease	400 (24%)	421 (32%) ^‡^	863 (47%) ^‡^
*Physical examination*			
Sinus rhythm	1474 (89%)	1203 (92%) ^‡^	1667 (91%) ^†^
Mean SBP levels (mm Hg)	119 ± 8.0	135 ± 3.1 ^‡^	153 ± 10.4 ^‡^
Number of SBP measurements	8487	6229	8792
Mean (SD) of SBP measurements	5.1 ± 3.3	4.8 ± 2.5 ^†^	4.8 ± 2.5 ^†^
Median (IQR) of SBP measurements	4 (3–5)	4 (3–5)	4 (3–6)
*Laboratory levels*			
Creatinine Clearance (mL/min)	80 ± 33	77 ± 29 ^†^	72 ± 29 ^‡^
Total cholesterol (mg/100 mL)	170 ± 35	177 ± 35 ^‡^	180 ± 37 ^‡^
LDL-cholesterol (mg/100 mL)	100 ± 30	104 ± 31 ^‡^	105 ± 32 ^‡^
*Drugs*			
Diuretics	513 (31%)	464 (36%) ^†^	931 (51%) ^‡^
Beta-blockers	907 (55%)	497 (38%) ^‡^	556 (30%) ^‡^
ACE-inhibitors	762 (46%)	536 (41%) *	872 (48%)
Angiotensin-II antagonists	299 (18%)	405 (31%) ^‡^	734 (40%) ^‡^
Calcium antagonists	321 (19%)	337 (26%) ^‡^	637 (35%) ^‡^
Antiplatelets	1478 (89%)	1185 (91%)	1630 (89%)
Anticoagulants	281 (17%)	176 (14%) *	262 (14%) *
Statins	1394 (84%)	1064 (82%)	1458 (80%) ^†^
Insulin	216 (13%)	169 (13%)	327 (18%) ^‡^
Oral antidiabetics	382 (23%)	415 (32%) ^‡^	611 (34%) ^‡^

Comparisons between patients with SBP levels <130 mm Hg: * *p* < 0.05; ^†^ *p* < 0.01; ^‡^ *p* < 0.001. Abbreviations: ACE, angiotensin-conversive enzyme; IQR: Interquartile range; LDL, low-density lipoprotein; SBP, systolic blood pressure; SD, standard deviation.

**Table 2:** Incidence rates (per 100 patient-years) of subsequent ischemic events and death, according to mean SBP levels during follow-up.

Subsequent Events	<130 mm Hg		130–140 mm Hg			>140 mm Hg		
	*n*	100 Patient-Years (95% CI)	*n*	100 Patient-Years (95% CI)	Rate Ratio (95% CI)	*n*	100 Patient-Years (95% CI)	Rate Ratio (95% CI)
*CAD patients, n*	900		442			380		
*Follow-up (years)*	1.406		0.574			0.486		
Myocardial infarction	32	2.27 (1.55–3.21)	18	3.13 (1.85–4.95)	1.37 (75–2.44)	27	5.55 (3.66–8.08)	2.44 (1.45–4.08) ^‡^
Ischemic stroke	2	0.14 (0.04–0.62)	6	1.05 (0.38–2.27)	7.34 (1.55–52.9) *	9	1.86 (0.85–3.51)	13.02 (3.10–88.41) ^‡^
Limb amputation	3	0.21 (0.05–0.58)	0	-	-	2	0.41 (0.46–1.48)	1.92 (0.22–1.29)
Death	28	1.99 (1.32–2.87)	23	4.01 (2.53–6.01)	2.01 (1.14–3.50) *	29	5.96 (3.99–8.57)	2.99 (1.77–5.06) ^‡^
*CVD patients, n*	364		439			580		
*Follow-up (years)*	0.571		0.580			0.689		
Myocardial infarction	4	0.71 (0.23–1.71)	3	0.52 (0.13–1.42)	0.73 (0.13–3.57)	3	0.44 (0.11–1.19)	0.62 (0.11–3.01)
Ischemic stroke	17	3.04 (1.83–4.76)	10	1.75 (0.89–3.12)	0.57 (0.25–1.26)	35	5.20 (3.68–7.16)	1.70 (0.96–3.11)
Limb amputation	2	0.35 (0.06–1.16)	0	-	-	2	0.29 (0.05–0.96)	0.82 (0.08–7.96)
Death	27	4.73 (3.18–6.79)	13	2.24 (1.25–3.73)	0.47 (0.23–0.91) *	28	4.07 (2.75–5.80)	0.85 (0.50–1.46)
*PAD patients, n*	400		421			863		
*Follow-up (years)*	0.578		0.637			1.352		
Myocardial infarction	17	2.98 (1.80–4.68)	10	1.58 (0.80–2.81)	0.53 (0.23–1.16)	22	1.65 (1.06–2.45)	0.55 (0.29–1.05)
Ischemic stroke	13	2.28 (1.27–3.79)	11	1.74 (0.92–3.03)	0.76 (0.33–1.73)	22	1.65 (1.06–2.45)	0.72 (0.36–1.47)
Limb amputation	23	3.97 (2.52–5.97)	16	2.56 (1.52–4.07)	0.63 (0.32–1.19)	26	1.92 (1.25–2.81)	0.48 (0.27–0.85) *
Death	47	8.13 (5.97–10.81)	25	3.92 (2.53–5.79)	0.48 (0.29–0.78) ^†^	40	2.95 (2.11–4.02)	0.36 (0.23–0.55) ^‡^

Comparisons between patients respect Systolic Blood Pressure < 130 mm Hg group: * *p* < 0.05; ^†^ *p* < 0.01; ^‡^ *p* < 0.001. Abbreviations: CI, confidence intervals. CAD, coronary artery disease; CVD, cerebrovascular disease; PAD, peripheral artery disease.

**Table 3:** Predictors of subsequent ischemic events. Multivariable analysis.

Predictors	CAD Patients	CVD Patients	PAD Patients
Age > 65 years	2.16 (1.17–4.01) *	-	-
Chronic heart disease	-	2.44 (1.13–5.28) *	-
SBP < 130 mm Hg	Ref.	Ref.	Ref. *
SBP 130–140 mm Hg	1.06 (0.62–1.80)	0.52 (0.25–1.10)	0.71 (0.46–1.09)
SBP >140 mm Hg	1.39 (0.86–2.26)	1.46 (0.84–2.54)	0.57 (0.39–0.83) ^†^
CrCl levels < 60 mL/min	2.19 (1.32–3.62) ^†^	-	-
Diuretics	1.83 (1.14–2.93) *	-	-
Beta-blockers	-	-	1.46 (1.03–2.09) *
Insulin	2.38 (1.53–3.70) ^‡^	-	2.83 (2.02–3.98) ^‡^
Antiplatelets	-	5.19 (1.94–13.9) ^†^	-
Anticoagulants	-	2.57 (1.45–4.56) ^†^	-

Comparisons: * *p* < 0.05; ^†^ *p* < 0.01; ^‡^ *p* < 0.001. Abbreviations: CAD, coronary artery disease; CrCl, creatinine clearance; CVD, cerebrovascular disease; PAD, peripheral artery disease; Ref, reference; SBP, systolic blood pressure. Variables included in the univariate analysis: the patient’s age, gender and body mass index, history of chronic heart disease, chronic lung disease, cancer, hypertension, diabetes or dyslipidemia, eGFR t presentation, family history of premature coronary artery disease, current smoking habit during follow-up, ABI, mean systolic blood pressure levels and heart rate during follow-up, and the use of beta-blockers, diuretics, calcium antagonists, ACE-inhibitors, angiotensins-II antagonists, oral antidiabetics, insulin therapy, statins anticoagulants or antiplatelets during follow-up.

**Table 4:** Predictors for mortality. Multivariate analysis.

Predictors	CAD Patients	CVD Patients	PAD Patients
Age > 65 years	3.42 (1.49–7.87) ^†^	3.66 (1.53–8.76) ^†^	1.74 (1.05–2.87) *
Cancer	2.33 (1.19–4.59) *	-	-
Chronic lung disease	1.85 (1.10–3.11) *	-	-
Chronic heart disease	1.86 (1.11–3.10) *	-	-
Sinus rhythm	-		0.27 (0.17–0.45) ^‡^
SBP < 130 mm Hg	Ref.	Ref. ^†^	Ref. ^‡^
SBP 130–140 mm Hg	1.28 (0.74–2.25)	0.39 (0.20–0.77) ^†^	0.47 (0.29–0.78) ^†^
SBP >140 mm Hg	1.46 (0.85–2.51)	0.46 (0.26–0.84) *	0.32 (0.21–0.50) ^‡^
CrCl levels < 60 mL/min	1.88 (1.07–3.28) *	1.90 (1.15–3.15) *	2.09 (1.35–3.26) ^†^
Beta-blockers	-	-	0.58 (0.35–0.98) *
Diuretics	1.98 (1.13–3.48) *	-	-
Statins	0.45 (0.27–0.75) ^†^	0.36 (0.22–0.59) ^‡^	0.39 (0.26–0.58) ^‡^
Insulin	-	1.85 (1.01–3.37) *	-

Comparisons: * *p* < 0.05; ^†^ *p* < 0.01; ^‡^ *p* < 0.001. Abbreviations: CAD, coronary artery disease; CrCl, creatinine clearance; CVD, cerebrovascular disease; PAD, peripheral artery disease; Ref, reference; SBP, systolic blood pressure. Variables included in the univariate analysis: the patient’s age, gender and body mass index, history of chronic heart disease, chronic lung disease, cancer, hypertension, diabetes or dyslipidemia, eGFR *t* presentation, family history of premature coronary artery disease, current smoking habit during follow-up, ABI, mean systolic blood pressure levels and heart rate during follow-up, and the use of beta-blockers, diuretics, calcium antagonists, ACE-inhibitors, angiotensins-II antagonists, oral antidiabetics, insulin therapy, statins anticoagulants or antiplatelets during follow-up.

## Appendix A

FRENA Investigators: Aguilar, E.; Alcalá-Pedrajas, J.N.; Alvarez, L.R.; Arnedo, G.; Coll, R.; García-Díaz, A.; López-Jiménez, L.; Monreal, M.; Pascual, M.T.; Sahuquillo, J.C.; Muñoz-Torrero, J.F.; Sanclemente, C.; Suriñach, J.M.; Toril, J.; Yeste. M.
